# Perceived Inequality and Wellness: Investigating the Longitudinal Links Between Relative Deprivation, Facets of Well-Being, and Self-Rated Health

**DOI:** 10.1007/s42761-025-00304-1

**Published:** 2025-05-09

**Authors:** Brian P. Don, Kieren J. Lilly, Chris G. Sibley, Nickola C. Overall, Danny Osborne

**Affiliations:** School of Psychology, University of Auckland, 23 Symonds St, Auckland, 1010 New Zealand

**Keywords:** Relative deprivation, Emotional well-being, Gratitude, Meaning in life, Belonging

## Abstract

Individual-based relative deprivation—feeling unfairly economically disadvantaged compared to others—fosters psychological ill-being and poorer physical health. However, research has overlooked the possibility that relative deprivation also undermines emotional *well-being*, which is distinct from ill-being and uniquely contributes to physical health. Using nine annual waves of a nationwide longitudinal panel sample of adults (*N* = 58,741–66,221), the current research utilized random intercept cross-lagged panel models to assess the between- and within-person longitudinal associations between individual-based relative deprivation and three key indicators of emotional well-being: gratitude, meaning in life, and belonging. Results indicated that individual-based relative deprivation longitudinally predicted lower gratitude, meaning in life, and belonging. Moreover, lower belonging (but not gratitude or meaning in life) mediated the longitudinal associations between greater individual-based relative deprivation and poorer physical health. These results suggest that individual-based relative deprivation undermines emotional well-being, which partially explains why relative deprivation correlates with poorer physical health.

**Supplementary Information:**

The online version contains supplementary material available at 10.1007/s42761-025-00304-1.

Income inequality—variability in economic resources across a particular region or population—remains a defining contemporary social challenge. Indeed, over the past several decades, income inequality within countries has increased in most parts of the world, especially within wealthy countries (OECD, 2011; Ortiz & Cummins, 2011; Saez & Zucman, 2016). Accordingly, global leaders have emphasized the costs of income inequality, and numerous economic and political organizations (e.g., the United Nations and the World Economic Forum; e.g., World Economic Forum, 2014) have identified income inequality as a critical social issue. And for good reason: extensive research demonstrates that societies with greater income inequality tend to have poorer health and well-being (see Pickett & Wilkinson, 2015, for a review).

Relative deprivation theory is one of the primary frameworks used to understand the influence of inequality on psychological and physical health. According to relative deprivation theory, perceptions of one’s own unfair economic disadvantage relative to others critically contribute to psychological and physical outcomes independent of actual levels of wealth (Smith et al., 2012; Walker & Smith, 2002). Relative deprivation theory incorporates both individual- and group-based relative deprivation (IRD and GRD, respectively), but our research focuses on how inequality contributes to psychological wellness at the individual level—outcomes that are unique to IRD (e.g., see Smith et al., 2012). Thus, we examine the longitudinal association between IRD—how much people perceive themselves as unfairly economically disadvantaged relative to (similar) others—and emotional well-being.

At the heart of relative deprivation is a social comparison, whereby people believe they are unjustly disadvantaged, resulting in feelings of anger and resentment that can accumulate to undermine mental and physical health (Smith et al., 2012; Smith & Huo, 2014). Indeed, numerous studies reveal that IRD correlates positively with psychological distress (Lilly et al., 2023; Mishra & Meadows, 2018; Osborne & Sibley, 2013) and symptoms of depression (e.g., Beshai et al., 2017; Qin et al., 2022), as well as maladaptive physical health outcomes, including worsened self-reported physical health, and physical health symptoms (Callan et al., 2015; Mishra & Carleton, 2015; Osborne et al., 2012).

Longitudinal research has been instrumental when examining how relative deprivation contributes to psychological outcomes (e.g., Lilly et al., 2023) because cross-sectional research cannot establish directionality between the variables of interest. Moreover, although numerous experimental studies have established causal relations between relative deprivation and key outcomes (Smith et al., 2012), these studies maximize internal validity and inference at the expense of understanding relative deprivation in the real world (i.e., ecological validity; Mitchell, 2012). Longitudinal designs strike a healthy balance between these two extremes by maintaining ecological validity while providing evidence of the directionality of associations. Longitudinal research is especially useful when examining the outcomes of relative deprivation because it can disaggregate within- and between-person processes (Curran & Bauer, 2011). Between-person associations refer to the stable, trait-like levels of relative deprivation that co-vary with trait-like levels of an outcome variable across time. In contrast, within-person associations reflect the relationship between departures from the trait-level mean at one assessment occasion and a given outcome at a subsequent assessment occasion. Without disaggregating between- and within-person associations, it is impossible to know whether within-person increases in relative deprivation are associated with within-person alterations in the psychological outcomes of interest (Curran & Bauer, 2011; Osborne & Little, 2024). Establishing this within-person association is critically important for showing that increases in relative deprivation are associated with subsequent decreases in psychological wellness *for individuals* (Curran & Bauer, 2011).

In this study, we utilized a large, multi-wave, longitudinal panel study to estimate a series of random intercept cross-lagged panel models (RI-CLPM) examining the longitudinal relationships between IRD and multiple measures of emotional well-being. The RI-CLPM was specifically designed to disaggregate between- and within-person associations in the context of a cross-lagged panel model by including correlated random intercepts (Hamaker et al., 2015; Osborne & Little, 2024). Thus, RI-CLPMs enable tests of the between- and within-person longitudinal associations between relative deprivation and emotional well-being while accounting for the bi-directional associations between these variables.

## Relative Deprivation and Psychological Wellness: A Missing Link

Although relative deprivation longitudinally contributes to *maladaptive* outcomes (e.g., depression; Beshai et al., 2017), prior research has scarcely examined how relative deprivation may longitudinally contribute to psychological *well-being*.^1^ This oversight matters because indicators of psychological wellness contribute to human health, vitality, and the promotion of a civil society *independent of* psychological ill-being (Frijters et al., 2020; Park et al., 2023; Pressman et al., 2019). To evaluate the longitudinal associations between relative deprivation and psychological wellness, we draw on recent theorizing on *emotional well-being*. Park et al (2023) argued that emotional well-being is a multidimensional concept that includes numerous affective and reflective features. Accordingly, the current study tests whether relative deprivation is longitudinally associated with multiple facets of emotional well-being.

Which facets of emotional well-being are especially relevant to IRD? We focused on gratitude, meaning in life, and belonging because each of these facets of emotional well-being are particularly relevant to the experience of relative deprivation. Gratitude refers to the extent to which people are appreciative and thankful, and is linked to (a) more helping behaviors (Bartlett & DeSteno, 2006), (b) behaviors that maintain and enhance relationships (Bartlett et al., 2012), and (c) better physical health (although not for all outcomes; see Jans-Beken et al., 2020). However, relative deprivation involves comparing what one has to other people, and judging that others have something better—a process likely to undermine one’s sense of appreciation, thereby longitudinally predicting lower gratitude.

Meaning in life is also an essential component of emotional well-being, and contributes to enhanced likeability (Stillman et al., 2011), lower cardiovascular risk (Kim et al., 2019), and reduced mortality (Boyle et al., 2009). By undermining one’s sense of fairness and trust, greater IRD may contribute to lower meaning in life. Across time, the feelings of unjust anger and resentment associated with relative deprivation are likely to reduce feelings of (a) purpose, (b) significance, and (c) coherence, all of which are key facets of meaning in life (Martela & Steger, 2016)*.*

Social well-being is also an important component of emotional well-being (Park et al., 2023). Indeed, the need to belong is a fundamental psychological need (Baumeister & Leary, 1995), and numerous models of well-being posit that healthy social relationships are essential to healthy psychological functioning (e.g., Ryan et al., 2008; Ryff, 2013). In the current study, we operationalize social well-being by focusing on feelings of belonging. IRD involves feelings of resentment towards others, which likely prompts social disconnection, thereby undermining felt belonging across time.

Notably, some authors (e.g., VanderWeele & Lomas, 2023) disagree with the structure of emotional well-being posited by Park et al. (2023). Moreover, gratitude, meaning in life, and belonging (a) do not fully encapsulate the concept of emotional well-being, which includes numerous other constructs, such as happiness, optimism, and other positive emotions (Park et al., 2023), and (b) are, to an extent, conceptually distinct constructs. Nonetheless, the three outcomes we have chosen are critical facets of emotional well-being and assess diverse but central components of the concept. Accordingly, while we acknowledge this study does not examine how IRD is associated with *all* aspects of emotional well-being, it provides an important initial examination of the between- and within-person longitudinal associations between IRD and key facets of emotional wellness.

## Emotional Well-Being as a Mechanism Linking Relative Deprivation to Health

Another goal of this study was to examine emotional well-being as a possible mediator by which IRD influences physical health. Prior research demonstrates that relative deprivation is associated with maladaptive physical health outcomes (Callan et al., 2015; Mishra & Carleton, 2015; Osborne et al., 2012), yet why these associations occur is less clear. An extensive body of research suggests that facets of emotional well-being—including gratitude, meaning in life, and social well-being—contribute to better physical health (Cohen et al., 2016; Hill & Turiano, 2014; Holt-Lundstad et al., 2010; Tugade et al., 2004). Accordingly, we test whether lower gratitude, meaning in life, and belonging partially explain the longitudinal association between IRD and physical health.

## Specifying the Hypothesized Causal Model

Recent reviews have argued that it is important for researchers using observational methods to be clear about the underlying causal question of interest (Grosz et al., 2020; Rohrer, 2018, 2024). That is, according to authors in this area (Grosz et al., 2020; Rohrer, 2018, 2024), although researchers using observational data often have a causal hypothesis in mind, researchers using observation data rarely clearly state that hypothesis, or discuss how their data or statistical methods help to evaluate causal claims. To address this issue, methodologists suggest using directed acyclic graphs, which are figures that depict the hypothesized causal associations among the primary variables of interest in a study (Rohrer, 2018, 2024). Importantly, given that covariates should also be carefully justified based on underlying causal claims and assumptions (Wysocki et al., 2022), covariates can be integrated into directed acyclic graphs.

**Fig. 1:**
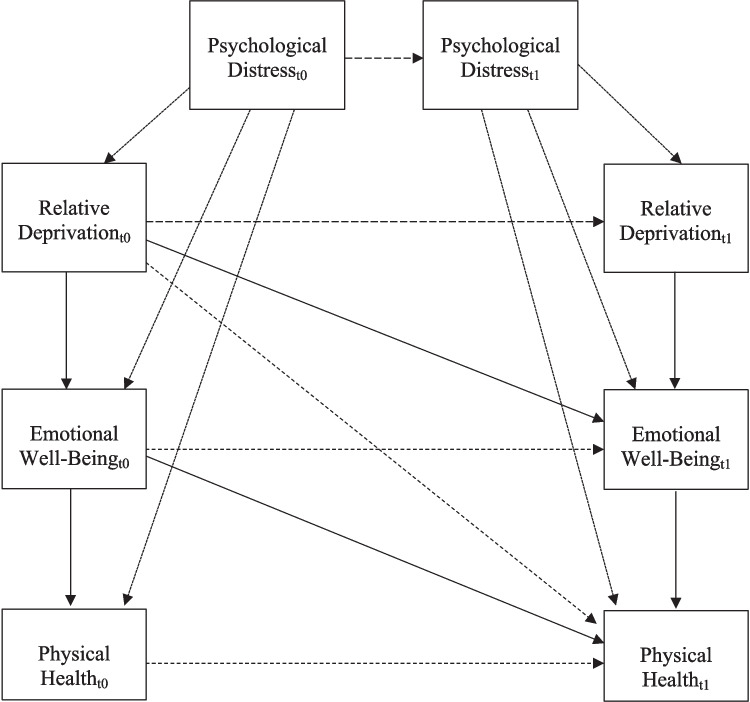
**Directed acyclic graph providing an overview of hypothesized causal associations among primary variables of interest.** This directed acyclic graph follows the recommendations of Rohrer (2018, 2024). We hypothesized that relative deprivation contributes to reductions in gratitude, meaning in life, and belonging (emotional well-being), each of which contributes to enhanced physical health. Psychological distress was examined as a confounding variable (i.e., a variable that can induce a non-casual association between relative deprivation, physical health, and the key mediators of interest). Focal hypothesized pathways are presented with solid lines

Figure 1 presents a directed acyclic graph providing an overview of the hypothesized causal associations among the primary variables in this study. Given that we explicitly used a multi-wave longitudinal study to examine between- and within-person associations, all our hypotheses are specified at both the concurrent and longitudinal levels. Our first and primary causal assumption is that greater relative deprivation undermines gratitude, meaning in life, and belonging (emotional well-being), both concurrently and longitudinally. Our second hypothesis is that reduced gratitude, meaning in life, and belonging meditate the associations between greater relative deprivation and reduced physical health.

As shown in Fig. 1, we account for one key confound in our causal model: psychological distress. Because prior research demonstrates that psychological distress is robustly associated with greater relative deprivation, lower physical emotional well-being, and lower physical health, we controlled for psychological distress to account for the possibility that it may be confounding the causal association between our variables of interest. Additionally, a critical component of our methodological approach is that we control for prior levels of each outcome variable when examining how each putative causal variable contributes to the outcome. For instance, when examining how relative deprivation potentially undermines gratitude, we statistically control for the association between prior levels of gratitude and subsequent levels of gratitude, thus isolating the unique effects of prior levels of relative deprivation on subsequent levels of relative deprivation (while removing the cross-sectional association between the two variables). Our chosen statistical framework—RI-CLPM—is ideally suited to test the hypothesized set of associations detailed in Fig. 1, including the cross-sectional, longitudinal, and mediational paths. We also pursue multiple robustness checks using other analytic techniques, including the stable trait, autoregressive trait, and state (STARTS) model (Kenny & Zautura, 2001) to overcome some of the limitations of the RI-CLPM (e.g., see Lucas, 2023).

It is important to note that there are other potential confounding variables that we were unable to assess and, thus, were not included in our statistical models or associated directed acrylic graph. For example, the association between relative deprivation and gratitude could be confounded by one’s level of exposure to media, which could contribute to increased relative deprivation and lower feelings of gratitude (e.g., Gkinopoulos et al., 2023). The ability to draw causal conclusions requires one to effectively close *all* potential backdoor paths (e.g., Rohrer, 2018), which is rarely possible in observational research. Thus, we note at the outset that our study is unable to provide definitive evidence of causality given we are not able to account for all possible backdoor paths via statistical covariation or random assignment. Our study does, however, provide foundations upon which future work can examine these questions.

## The Current Study

Using a longitudinal, nationwide random sample of adults, we tested whether (a) IRD longitudinally predicts lower emotional well-being, and (b) emotional well-being partially mediates the longitudinal association between relative deprivation and poorer physical health. We also ran additional tests accounting for psychological distress to illustrate that the longitudinal associations between relative deprivation, emotional well-being, and health were independent of distress. Finally, we ran a series of robustness checks to examine the replicability of our results across different model specifications.

## Method

### Participants and Procedures

We used data from Waves 5 (2013) through 13 (2021) of the New Zealand Attitudes and Values Study (NZAVS). The New Zealand Attitudes and Values Study was approved by the University of Auckland Human Participants Ethics Committee. All participants provided informed consent to participate in this research, and this the study was performed in accordance with the ethical standards outlined in the 1964 Declaration of Helsinki. Wave 5 was the first year to assess self-rated health. Because meaning in life and gratitude were only assessed at later waves of data collection (Waves 10 to 13), those analyses involve fewer data points. The year of data collection and sample sizes were as follows: Wave 5 (2013, *N* = 18,261), Wave 6 (2014, *N* = 15,820), Wave 7 (2015, *N* = 13,942), Wave 8 (2016, *N* = 21,936), Wave 9 (2017, *N* = 17,072), Wave 10 (2018, *N* = 47,951), Wave 11 (2019, *N* = 42,684), Wave 12 (2020, *N* = 38,550), and Wave 13 (2021, *N* = 34,131). Supplemental Table 1 displays participant demographic information.

Information on the sample procedure and retention of participants across the course of the NZAVS can be found on the OSF page for the NZAVS here: https://osf.io/75snb/. Measures and syntax for this specific paper can be found here: https://osf.io/x3km5/?view_only=a80e0ca2fe8e49d38dd660b690341ec6. Because not all participants returned for follow-ups at each of the waves, we estimated all models using full information maximum likelihood (FIML) to address missing data. This estimation method enables the use of all available data, including participants who completed the variables of interest across all of the longitudinal waves (i.e., those who had no missing data), and participants who provided partial or complete responses at any of the waves (i.e., those who had some missing data), while reducing bias that may be introduced by other methods of dealing with missing cases (e.g., listwise deletion; see Enders & Bandalos, 2001).

### Measures

Measures were embedded in a large omnibus questionnaire that included additional questions outside the scope of the current study. Unless noted, participants responded on a 1 (*Strongly Disagree*) to 7 (*Strongly Agree*) scale. Supplemental Table 2 displays the internal consistency of all measures used in this research. Sibley et al. (2025) recently validated the short-form measures used in the NZAVS by showing that these scales have acceptable levels of reliability relative to their long-form counterparts.

#### Relative Deprivation

IRD was assessed using two items adapted from Abrams and Grant (2012). Participants were presented with the items: “*I’m frustrated by what I earn relative to other people in New Zealand*” and “*I generally earn less than other people in New Zealand*”.

#### Gratitude

Three items from the Gratitude Questionnaire (McCullough et al., 2002) were administered. Items assessed the extent to which participants generally experienced gratitude throughout their life: “*I have much in my life to be thankful for*”, “*When I look at the world, I don’t see much to be grateful for*”, and “*I am grateful to a wide variety of people*”.

#### Meaning in Life

Meaning in life was assessed using two items from the Meaning in Life Questionnaire (Steger et al., 2006): “*My life has a clear sense of purpose*” and “*I have a good sense of what makes my life meaningful*”.

#### Belonging

Belonging was assessed using three items adapted from Hagerty and Patusky (1995): “(I) *Know that people in my life accept and value me*”, “(I) *Feel like an outsider”*, and “(I) *Know that people around me share my attitudes and beliefs*”. Participants responded to the items on a 1 (*Very Inaccurate*) to 7 (*Very Accurate*) scale.

#### Self-Rated Health

Participants completed three items (e.g., “*In general, would you say your health is…*”) from the Short-Form Subjective Health Scale (Ware & Sherbourne, 1992). Participants responded on a 1 (*Poor*) to 7 (*Excellent*) scale.

#### Psychological Distress

Participants completed the K6 psychological distress scale (Kessler et al., 2010). Participants responded to six items (e.g., *During the last 30 days, how often did you feel worthless*?) on a 0 (*None of the Time*) to 4 (*All of the Time*) scale.

### Data Analytic Strategy

**Fig. 2:**
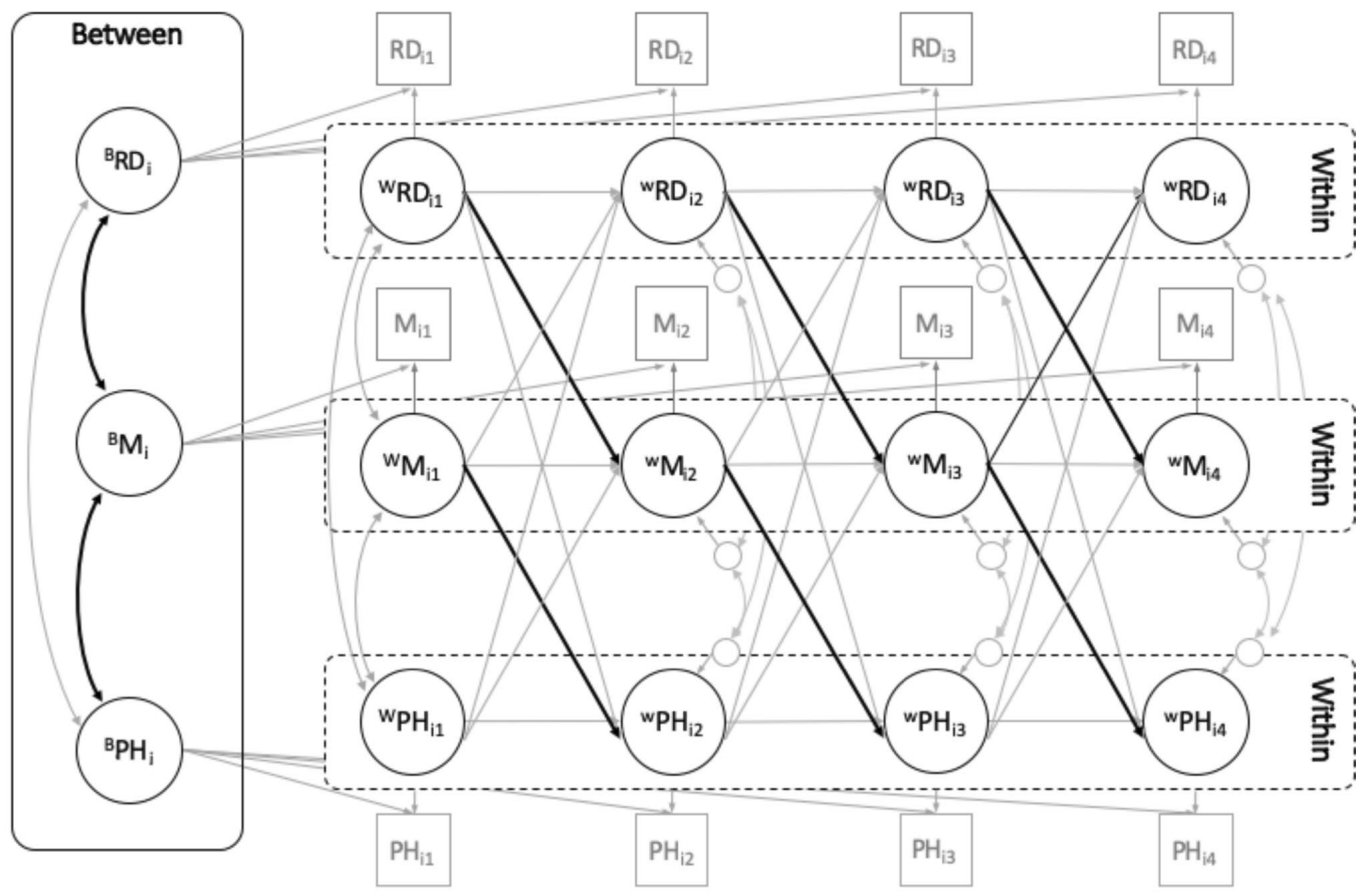
**Detailed overview of specification of RI-CLPMs in primary analyses.** RD, relative deprivation; M, meaning in life; PH, self-rated physical health. An identical model was estimated for gratitude as a mediator with gratitude replacing meaning in life in the model. The model containing belonging as a mediator was estimated similarly, but used nine waves of data. Focal pathways are presented in bold (i.e., those in which relative deprivation predicts emotional well-being, and emotional well-being predicts physical health)

We analyzed our data using a series of RI-CLPMs because this analytic approach closely matched the underlying causal structure of the hypotheses outlined in Fig. 1. Specifically, our hypotheses assume that relative deprivation contributes to reductions in emotional well-being and, therefore, decrements in physical health across time. RI-CLPM represents an excellent way to test these hypotheses with multi-wave, longitudinal data. We, therefore, estimated a series of RI-CLPMs in MPlus version 8.9 to examine whether (a) relative deprivation predicted reduced psychological well-being across time and (b) reduced psychological well-being mediated the longitudinal links between relative deprivation and self-rated physical health***.*** Compared to other methods of examining longitudinal panel data, a key advantage of the RI-CLPM is that it allows for an examination of lagged associations while disaggregating between- and within-person associations (Hamaker et al., 2015; Osborne & Little, 2024). Moreover, RI-CLPMs do not require intensive longitudinal data (Orth et al., 2021; Osborne & Little, 2024) and are therefore capable of examining longitudinal change with as few as three (or four) assessment occasions (as is the case here). Figure 2 presents how the RI- CLPMs were specified for these analyses, using the example of meaning in life as the mediator between relative deprivation and self-rated physical health.

All models tested the longitudinal, lagged associations between relative deprivation, emotional well-being variables (either meaning, gratitude, belonging, or sense of community), and self-rated health. In each model, we utilized the indirect command in MPlus to test the significance of the indirect effect of relative deprivation $\rightarrow$ emotional well-being $\rightarrow$ self-rated health. Although we had multiple waves of data (nine waves for belonging, and four waves for gratitude and meaning in life), our primary interest was in the overall association between relative deprivation and the subsequent wave of emotional well-being *regardless* of the specific wave of data collection. We also had no theoretical reason to believe that the strength of the associations between the variables of interest would vary across the waves of the study, and, thus, assumed stationarity across assessment occasions (see Orth et al., 2021). Accordingly, the unstandardized regression coefficients provide an overall, average estimate of the longitudinal association between variables at the prior time point and the subsequent assessment (as opposed to separate estimates of the association between the predictor and outcome at each prior and subsequent wave; Orth et al., 2021). Each model also provided the overall average estimate of the indirect effect of interest. To assess the size of the cross-lagged paths, we examined the size of the standardized coefficients according to the guidelines for RI-CLPM provided by Orth et al (2024): 03 (small), 0.07 (medium), and 0.12 (large).

We note that RI-CLPMs are particularly useful in this context because they also estimate the possibility of reverse directionality. Because IRD captures *perceptions* of inequality, and inequality is associated with poorer mental well-being (see Osborne et al., 2022), decrements to mental well-being could influence subsequent levels of IRD (see Smith et al., 2012). Our RI-CLPMs examine the possibility that prior well-being predicts subsequent perceptions of relative deprivation by testing whether IRD is associated with reduced meaning in life, gratitude, and belonging. Moreover, given that our supplemental models account for both psychological wellness and psychological distress, our models represent a rigorous and tightly controlled longitudinal examination of whether IRD longitudinally contributes to emotional well-being, even after accounting for how mental distress and well-being may contribute to IRD.

We also estimated three separate sets of alternative models to investigate the robustness of our focal analyses. First, traditional RI-CLPMs include autoregressive paths only for the immediately preceding wave of data. Yet unknown factors that are present at other preceding waves of data collection may also impact the results (see Little, 2024). Given that these unmeasured time-varying confounds can impact the associations revealed in panel designs (e.g., see Rohrer & Murayama, 2023), we followed Lüdtke and Robitzsch’s (2022) approach and also estimated models that included autoregressive paths with two lags (i.e., AR2 s; for example, an autoregressive effect of IRD at Wave 5 on IRD at Wave 7). Second, as is typical in RI-CLPMs, our primary models assessed each variable using scale means of the overall construct (Hamaker et al., 2015). As such, our primary RI-CLPMs do not take into account the likelihood that our constructs were measured imprecisely. To account for measurement error, we ran additional analyses estimating multiple-indicator RI-CLPMs by treating each construct (e.g., IRD, meaning in life, and self-rated health) at each measurement occasion as latent factors. Although this approach is computationally difficult, it adjusts for measurement error in the focal constructs. Finally, some have argued that the STARTS model represents a superior alternative to the RI-CLPM because it estimates, and therefore accounts for, measurement error of the focal constructs (Lucas, 2023). Accordingly, we reconducted all our primary analyses using the STARTS model to see if the substantive conclusions changed after acknowledging these parameters that are unaccounted for in an RI-CLPM. Thus, our robustness checks seek to replicate our primary analyses using three separate extensions of, and alternatives to, the traditional RI-CLPM.

## Results

### Gratitude

Table 1 summarizes the RI-CLPM examining the link between IRD, gratitude, and self-rated health across four waves of data. Between-person associations indicated that people who, on average, reported higher IRD across assessment occasions also reported lower gratitude and self-rated physical health across assessment occasions. People who, on average, reported greater gratitude across assessment occasions also reported better physical health.

**Table 1:** Results of RI-CLPM for gratitude

		95% CI				
*Between-person*	*B*	*LL*	*UL*	*p*		
Relative deprivation ⟷ gratitude	** − .33**	** − .28**	** − .26**	** < .001**		
Relative deprivation ⟷ physical health	** − **.32	** − **.39	** − **.36	< .001		
Gratitude ⟷ physical health	**.37**	**.24**	**.26**	** < .001**		
*Within-person*				95% CI		
Outcome_T_	Predictor_T-1_	*B*	*SE*	*LL*	*UL*	*p*
Relative deprivation	Gratitude	**−**.01	.01	**−**.03	.00	.116
	Self-rated health	**−**.02	.01	**−**.03	.00	.061
	Relative deprivation	.12	.01	.11	.14	< .001
Gratitude	Gratitude	.06	.01	.05	.07	< .001
	Self-rated health	.01	.01	.00	.02	.062
	**Relative deprivation**	**− .01**	**.00**	**− .02**	**.00**	**.001**
Self-rated health	Gratitude	.00	.01	**−**.01	.01	.728
	Self-rated health	.13	.01	.12	.14	< .001
	Relative deprivation	.00	.00	**−**.01	.00	.345

*n *= 58,741. Paths in bold represent statistically significant predicted pathways. Model fit indices: *χ*^2^_(39)_ = 366.42, *p* < .001; CFI = 0.998, RMSEA = 0.012 [0.011, 0.013], *p* > .999; SRMR = 0.017

Results for the within-person associations partially supported the expected longitudinal associations. Increases from a person’s usual levels of IRD at one assessment occasion predicted declines from their typical level of gratitude at the subsequent assessment—even after controlling for departures from their usual levels of gratitude from the prior wave (prior wave gratitude to subsequent wave gratitude *B* = 0.06). The size of the within-person, cross-lagged association was small (*B* = − 0.02). Controlling for the prior wave of self-rated health, departures from a person’s trait-level gratitude were not associated with their subsequent reports of self-rated health. Thus, the *a*, but not the *b*, path underlying the hypothesized within-person indirect effect of relative deprivation on self-rated health via gratitude was statistically significant. Accordingly, the indirect effect was not significant, *b*_indirect_ = .001, *SE* = .001, *p* = .73. An ancillary analysis controlling for psychological distress yielded conclusions identical to those presented in Table 1 (see Supplemental Table 3).

### Meaning in Life

Table 2 summarizes the RI-CLPM examining the links between IRD, meaning, and self-rated health across four annual assessments. At the between-person level, results were consistent with expectations: people who reported greater IRD across assessment occasions reported lower meaning in life and worse physical health. People who reported greater meaning in life across assessments also reported better physical health compared to those who reported lower meaning in life.

**Table 2:** Results of RI-CLPM for meaning in life

		95% CI				
*Between-person*	*B*	*LL*	*UL*	*p*		
Relative deprivation ⟷ meaning in life	** − .37**	** − .38**	** − .35**	** < .001**		
Relative deprivation ⟷ physical health	** − **.38	** − **.39	** − **.36	< .001		
Meaning in life ⟷ physical health	**.40**	**.39**	**.41**	** < .001**		
*Within-person*				95% CI		
Outcome_T_	Predictor_T-1_	*B*	*SE*	*LL*	*UL*	*p*
Relative deprivation	Meaning in life	**−**.03	.01	**−**.04	**−**.01	< .001
	Self-rated health	**−**.01	.01	**−**.03	.01	.178
	Relative deprivation	.12	.01	.11	.14	< .001
Meaning in life	Meaning in life	.12	.01	.11	.14	< .001
	Self-rated health	.02	.01	.01	.03	.002
	**Relative deprivation**	**− .01**	**.00**	**− .02**	**.00**	**.023**
Self-rated health	Meaning in life	.01	.01	.00	.02	.186
	Self-rated health	.13	.01	.12	.14	< .001
	Relative deprivation	.00	.00	**−**.01	.00	.486

*n* = 58,741. Paths in bold represent statistically significant predicted pathways. Model fit indice: *χ*^2^_(39)_ = 474.92, *p* < .001; CFI = 0.998, RMSEA = 0.014 [0.013, 0.015], *p* > .999; SRMR = 0.019

Turning to the within-person associations, results again provided partial support for our hypotheses. Increases from a person’s usual levels of IRD at one assessment occasion predicted time-specific declines in their typical level of meaning in life at the subsequent assessment—even after controlling for prior departures in meaning in life (prior wave meaning to subsequent wave meaning *B* = .13). The size of the focal cross-lagged association was small (*B* = − .01). Controlling for prior departures in self-rated health, departures from a person’s usual levels of meaning in life were not associated with their subsequent reports of self-rated health (*B* = .01). As such, because the *a*, but not the *b*, path was again not statistically significant, the indirect effect was also not statistically significant, *b*_indirect_ = .001, SE = .001, *p* = .26. An ancillary analysis controlling for psychological distress yielded one important change to these results: the within-person link between IRD and subsequent declines in meaning in life became non-significant (see Supplemental Table 4). Thus, the longitudinal within-person association between relative deprivation and meaning in life was unreliable after accounting for psychological distress. All other results remained unchanged.

### Belonging

Table 3 summarizes our RI-CLPM examining the link between IRD, belonging, and self-rated health across nine annual assessments. At the between-person level, results were consistent with our expectations: people who reported greater relative deprivation across assessments reported lower belonging and worse physical health. People who, on average, reported greater belonging also reported better physical health across assessment occasions.

**Table 3:** Results of RI-CLPM for belonging

		95% CI				
*Between-person*	*B*	*LL*	*UL*	*p*		
Relative deprivation ⟷ belonging	** − .38**	− **.39**	− **.37**	** < .001**		
Relative deprivation ⟷ physical health	− .37	− .38	− .35	< .001		
Belonging ⟷ physical health	**.35**	**.35**	**.36**	** < .001**		
*Within-person*				95% CI		
Outcome_T_	Predictor_T-1_	*B*	*SE*	*LL*	*UL*	*p*
Relative deprivation	Belonging	− .01	.01	− .02	.00	.029
	Self-rated health	− .01	.01	− .02	.00	.004
	Relative deprivation	.17	.00	.16	.18	< .001
Belonging	Belonging	.12	.00	.12	.13	< .001
	Self-rated health	.03	.00	.02	.03	< .001
	**Relative deprivation**	−**0.01**	**.00**	−**.01**	−**.01**	**< .001**
Self-rated health	**Belonging**	**.02**	**.00**	**.01**	**.02**	**< .001**
	Self-rated health	.18	.00	.18	.19	< .001
	Relative deprivation	− .01	.00	− .01	.00	.007

*n* = 66,221. Paths in bold represent statistically significant predicted pathways. Model fit indices: *χ*^2^_(309)_ = 3225.359, *p* < .001; CFI = 0.993, RMSEA = 0.012 [0.012, 0.012], *p* > .999; SRMR = 0.027

Focusing on the within-person associations, results supported our hypotheses. Increases from a person’s usual levels of IRD at one assessment occasion predicted time-specific declines in their typical level of belonging at the subsequent assessment—even after controlling for prior departures in belonging (prior wave belonging to subsequent wave belonging *B* = .12). The size of this within-person, cross-lagged association was small (*B* = − .02). Moreover, after controlling for prior departures in self-rated health, increases in a person’s usual levels of belonging at one wave predicted time-specific increases in their typical level of self-rated health at the subsequent assessment. The size of this association was small (*B* = .02). Also consistent with expectations, the within-person indirect association between relative deprivation and self-rated health via belonging was statistically significant, *b*_indirect_ = .001, *SE* = .001, *p* = .002. An ancillary analysis controlling for psychological distress yielded substantively similar between- and within-person pathways (see Supplemental Table 5). Thus, consistent with expectations, even after controlling for psychological distress, (a) greater relative deprivation predicted lower belonging, (b) lower belonging predicted worse physical health, and (c) both of these associations occurred at both the between- and within-person levels.

### Supplementary Analyses

We conducted a series of additional analyses to check the robustness of our primary models, including a set of (a) RI-CLPMs with AR2 s, (b) multiple-indicator RI-CLPMs, and (c) STARTS models re-testing our primary hypotheses. Results of these models are presented in the supplemental materials (see Supplemental Tables 6, 7, 8, 9, 10, 11, 12, 13 and 14) and are largely consistent with the results of our primary analyses, with two exceptions. First, in the multiple-indicator RI-CLPM examining the indirect association between IRD and self-rated health via belonging (see Supplemental Table 11), all results were consistent with our primary analyses except for the longitudinal, within-person association between belonging and self-rated health. Specifically, greater belonging was associated with *worse* self-rated physical health at the within-person level. Given that this finding is inconsistent with (a) the other supplemental analysis, (b) our primary analyses, and (c) a plethora of literature demonstrating the importance of belonging and social relatedness to physical health (e.g., Holt-Lundstad et al., 2010), we think this finding should be treated with caution. Additionally, in the STARTS models (see Supplemental Tables 12, 13 and 14), all results were identical to our focal analyses except for the within-person associations between greater relative deprivation and subsequent declines in gratitude. Specifically, this within-person association was non-significant in the STARTS model.

## Discussion

Using a nationwide random sample of adults, we extended prior research by examining the between- and within-person longitudinal associations between IRD, emotional well-being, and self-rated physical health. Results from a series of RI-CLPMs indicated that, at the between-person level, relative deprivation was associated with lower gratitude, meaning in life, and belonging even when controlling for differences in psychological distress. At the within-person level, IRD predicted lower gratitude and belonging across time, but not lower meaning in life, after controlling for distress. The hypothesized longitudinal indirect effect of relative deprivation lower well-being worse physical health only emerged for belonging. The implications of these results are discussed below.

### Understanding the Longitudinal Association Between Relative Deprivation and Emotional Well-Being

We expand the principal focus of the associations between IRD and psychological ill-being (e.g., see Osborne & Sibley, 2013) by demonstrating that relative deprivation is longitudinally associated with two key facets of emotional well-being—gratitude and belonging —even when accounting for psychological distress. These results have important theoretical implications for the relative deprivation literature because (a) extensive research demonstrates that psychological well-being and ill-being are distinct, both in form and function (e.g., Fredrickson, 2013; Frijters et al., 2020; Park et al., 2023), and (b) little prior work has linked relative deprivation to aspects of psychological well-being (none of which was longitudinal). Additionally, although the size of the cross-lagged associations between IRD and subsequent emotional well-being were small, they were notable in that they (a) emerged after controlling for the large associations between prior wave and subsequent wave levels of the outcome variable and (b) were similar in size to the well-established link between IRD and subsequent levels of psychological distress (e.g., *B* = 0.014 from the model examining IRD belonging physical health while controlling for psychological distress). Thus, the current results offer important new evidence that emotional and social well-being should be considered key outcomes of IRD alongside psychological distress.

Notably, relative deprivation was especially relevant to gratitude and belonging, but less robustly predicted meaning in life when adjusting for psychological distress. Gratitude represents the tendency for people to consciously appreciate the positive aspects of life (McCullough et al., 2002), and the social comparison associated with relative deprivation appears to alter people’s views of these positive aspects, reducing feelings of being grateful for what they have. Similarly, our results suggest that IRD is especially relevant to feelings of belonging: relative deprivation theoretically involves direct social comparisons and angry resentment (Smith & Pettigrew, 2014), which appear to erode feelings of social connection.

It is important to return to the casual assumptions underlying our hypotheses outlined in Fig. 1. We proposed that relative deprivation undermines positive aspects of emotional well-being concurrently and across time, including gratitude, meaning in life, and belonging, even when controlling for psychological distress. Our analyses are high-powered, longitudinal, and control for key confounds (psychological distress and bi-directionality among key variables of interest). Thus, the results provide initial evidence consistent with our underlying assumption that relative deprivation may undermine gratitude and belonging (but not meaning in life). Yet, as noted by Rohrer (2018) and others, drawing causal conclusions from observational data requires effectively controlling for all possible confounds, and making numerous other assumptions about the nature of the data (e.g., no measurement error is present), none of which we could do here. Thus, while we provide initial evidence in support of the hypothesized association between relative deprivation and reduced gratitude and belonging, future research that uses a combination of carefully controlled observational and experimental work is needed to replicate and extend our work.

### Relative Deprivation and Physical Health: Emotional Well-Being as a Mediator

The second aim of our study was to examine whether emotional well-being helped explain how relative deprivation may influence physical health. Within-person tests of indirect effects between relative deprivation and physical health via emotional well-being emerged for belonging, even when controlling for psychological distress. These results suggest that IRD may contribute to physical health by undermining a critical facet of social well-being—belonging—consistent with extensive evidence that healthy social relationships are vital to physical health (e.g., Holt-Lunstad et al., 2010). We also note the important caveats related to our underlying causal assumptions: although we found evidence consistent with the hypothesized causal relationship summarized in Fig. 1, we were unable to control for every possible confound, or perfectly measure all variables in this research (among other limitations). As such, this work provides only preliminary evidence of the hypothesized indirect association between IRD and physical health via belonging.

Although the within-person longitudinal association between IRD and gratitude was significant, the within-person association between gratitude and self-rated health was not. Thus, gratitude did not mediate the longitudinal association between relative deprivation and physical health. Nevertheless, gratitude has numerous important benefits independent of physical health (Bartlett & DeSteno, 2006; Bartlett et al., 2012; DeSteno et al., 2019). Thus, despite the fact that we did not observe the expected association between IRD and physical health via gratitude, the longitudinal link between relative deprivation and lower gratitude has numerous important implications for the behavior of individuals who perceive greater inequality.

We also note that ancillary analyses using the STARTS model failed to identify a within-person longitudinal association between greater relative deprivation and lower gratitude. Although we are generally confident in the results from our focal RI-CLPMs, given that they were largely consistent across many different model specifications, it is important to interpret the within-person longitudinal link between greater relative deprivation and lower gratitude with caution given the results of the STARTS models. Results from the STARTS models do, however, provide further verification for the between- and within-person associations between greater relative deprivation and lower belonging. Indeed, these associations were significant in every model that we tested regardless of the model specification or analytic approach.

### Caveats and Conclusions

We drew on a highly-powered, nationwide random sample of adults to assess within-person longitudinal changes. These strengths are accompanied by limitations, such as the use of self-reports, and the availability of only three measures of emotional well-being. New Zealand also has lower current levels of inequality than countries like the USA and the UK (OECD, 2023). Our novel results nevertheless highlight the importance of future research examining whether relative deprivation undermines other aspects of psychological wellness, and whether the experience and effects of relative deprivation vary across countries. Indeed, the current study demonstrated for the first time that (a) greater relative deprivation is longitudinally associated with within-person reductions in gratitude and belonging and (b) belonging partially mediates the links between relative deprivation and physical health. This work highlights the importance of psychological wellness in understanding inequality, which remains one of society’s greatest challenges.

## Supplementary Information

Below is the link to the electronic supplementary material.

ESM 1 (DOCX 69.3 KB)

## Footnotes

1 Although limited research has examined how relative deprivation contributes to psychological well-being, some research has examined how income inequality (rather than perceived inequality) contributes to aspects of well-being. Research in this area suggests that greater income inequality at the societal level tends to be negatively associated with psychological well-being (Cheung & Lucas, 2016; Oishi & Kesebir, 2015; Oishi et al., 2011). However, this association may vary depending on stage of economic development. For example, in the early stages of economic development, higher income inequality may be positively related to facets of well-being, such as feelings of hope (Cheung, 2016; Cheung & Lucas, 2020).

## Additional Information

### Data Availability

No datasets were generated or analysed during the current study.
