# Supplementary material for: Perceived Inequality and Wellness: Investigating the Longitudinal Links Between Relative Deprivation, Facets of Well-Being, and Self-Rated Health
Source: Affect Sci. 2025 May 9;6(2):308–20. doi: 10.1007/s42761-025-00304-1 (PMC12209158; doi:10.1007/s42761-025-00304-1)
Supplement: Supplementary file 1 — (DOCX 69.3 KB) [file 42761_2025_304_MOESM1_ESM.docx]

**Supplemental Table 1**

*Demographic information for participants in the NZAVS study*

|  | | *Wave 5* | *Wave 6* | *Wave 7* | *Wave 8* | *Wave 9* | *Wave 10* | *Wave 11* | *Wave 12* | *Wave 13* |
| --- | --- | --- | --- | --- | --- | --- | --- | --- | --- | --- |
| Sample Size | | 18,261 | 15,820 | 13,942 | 21,936 | 17,072 | 47,948 | 42,681 | 38,550 | 34,131 |
| Retention from Prior Year | | - | 81.47% | 79.33% | 85.59% | 71.96% | 82.30% | 72.54% | 78.06% | 74.29% |
| Age *M* | | 48.14 | 49.83 | 51.3 | 50.12 | 51.83 | 49.09 | 52.05 | 53.45 | 54.89 |
| Age *SD* | | 14.07 | 14.03 | 13.9 | 13.92 | 13.77 | 13.86 | 13.87 | 13.69 | 13.66 |
| Gender | |  |  |  |  |  |  |  |  |  |
|  | Women | 11,442 | 6,661 | 8,712 | 13,719 | 10,787 | 29,958 | 27,176 | 24,528 | 21,787 |
|  | Men | 6,785 | 5,799 | 5,188 | 8,158 | 6,230 | 17,783 | 15,238 | 13,796 | 12,113 |
|  | Gender Diverse | 34 | 30 | 33 | 65 | 55 | 207 | 269 | 226 | 231 |
| Ethnicity | |  |  |  |  |  |  |  |  |  |
|  | NZ Euro/Pākehā | 15,607 | 14,161 | 12,506 | 19,478 | 15,606 | 42,543 | 39,525 | 35,543 | 31,534 |
|  | Māori | 2,328 | 1,977 | 1,672 | 2,473 | 2,007 | 4,696 | 4,314 | 3,374 | 3,036 |
|  | Pasifika | 625 | 526 | 427 | 574 | 466 | 1,039 | 1,148 | 857 | 594 |
|  | Asian | 814 | 684 | 545 | 1001 | 700 | 2,541 | 1,900 | 1,558 | 1,231 |
| Born in NZ | | 79.11 | 79.60 | 79.88 | 77.68 | 79.61 | 77.86 | 78.18 | 78.52 | 78.61 |
| Household income (NZD) | | 101,732 | 104,643 | 107,139 | 108,600 | 113,643 | 115,061 | 117,971 | 120,782 | 125,472 |

*Note*. Retention from Prior Year refers to the percentage of participants who returned for follow-up from the prior year’s sample.

**Supplemental Table 2**

*Cronbach’s Alphas for primary study variables*

| *Variable* | *Wave 5* | *Wave 6* | *Wave 7* | *Wave 8* | *Wave 9* | *Wave 10* | *Wave 11* | *Wave 12* | *Wave 13* |
| --- | --- | --- | --- | --- | --- | --- | --- | --- | --- |
| Relative Deprivation | 0.60 | 0.58 | 0.59 | 0.60 | 0.59 | 0.58 | 0.59 | 0.59 | 0.59 |
| Gratitude | - | - | - | - | - | 0.53 | 0.54 | 0.56 | 0.57 |
| Meaning in Life | - | - | - | - | - | 0.72 | 0.74 | 0.75 | 0.75 |
| Belonging | 0.58 | 0.60 | 0.61 | 0.58 | 0.60 | 0.58 | 0.60 | 0.64 | 0.60 |
| Self-Rated Health | 0.61 | 0.61 | 0.60 | 0.58 | 0.60 | 0.58 | 0.62 | 0.61 | 0.63 |
| Psychological Distress | 0.84 | 0.85 | 0.85 | 0.85 | 0.85 | 0.85 | 0.86 | 0.86 | 0.86 |

*Note*. Gratitude and meaning in life were not assessed at Waves 5 through 9.

**Supplemental Table 3**

*Results of RI-CLPM for Gratitude while Controlling for Psychological Distress*

|  |  | 95% CI | |  |
| --- | --- | --- | --- | --- |
| *Between-Person Effects* | *B* | *LL* | *UL* | *p* |
| Relative Deprivation ⟷ Gratitude | -0.27 | -0.28 | -0.26 | <.001 |
| Relative Deprivation ⟷ Physical Health | -0.38 | -0.39 | -0.37 | <.001 |
| Relative Deprivation ⟷ Psychological Distress | 0.28 | 0.28 | 0.29 | <.001 |
| Gratitude ⟷ Physical Health | 0.25 | 0.24 | 0.26 | <.001 |
| Gratitude ⟷ Psychological Distress | -0.16 | -0.17 | -0.16 | <.001 |
| Physical Health ⟷ Psychological Distress | -0.29 | -0.29 | -0.28 | <.001 |

|  |  |  |  | 95% CI | |  |
| --- | --- | --- | --- | --- | --- | --- |
| Outcome_T_ | Predictor_T-1_ | *B* | *SE* | *LL* | *UL* | *p* |
| Relative | Gratitude | -0.01 | 0.01 | -0.03 | 0.004 | .138 |
| Deprivation | Self-rated health | -0.009 | 0.01 | -0.03 | 0.01 | .250 |
|  | Relative deprivation | 0.12 | 0.01 | 0.11 | 0.13 | <.001 |
|  | Psychological distress | 0.04 | 0.01 | 0.01 | 0.06 | .008 |
| Gratitude | Gratitude | 0.06 | 0.01 | 0.05 | 0.07 | <.001 |
|  | Self-rated health | 0.003 | 0.01 | -0.006 | 0.01 | .499 |
|  | Relative deprivation | -0.009 | 0.003 | -0.02 | -0.003 | .002 |
|  | Psychological distress | -0.056 | 0.01 | -0.08 | -0.04 | <.001 |
| Health | Gratitude | 0.001 | 0.01 | -0.01 | 0.01 | .891 |
|  | Self-rated health | 0.13 | 0.01 | 0.11 | 0.14 | <.001 |
|  | Relative deprivation | -0.002 | 0.004 | -0.01 | 0.01 | .516 |
|  | Psychological distress | -0.03 | 0.01 | -0.05 | -0.01 | .002 |
| Psychological | Gratitude | -0.008 | 0.004 | -0.02 | -0.001 | .026 |
| Distress | Self-rated health | -0.01 | 0.003 | -0.02 | -0.008 | <.001 |
|  | Relative deprivation | 0.004 | 0.002 | 0.00 | 0.01 | .043 |
|  | Psychological distress | 0.11 | 0.01 | 0.09 | 0.12 | <.001 |

*Note*. *n* = 58,742. Model fit indices: χ2 (70) = 1006.07, p < .001; CFI = 0.997, RMSEA = 0.015 [0.014, 0.016], p > .999, SRMR = 0.017.

**Supplemental Table 4**

*Results of RI-CLPM for Meaning in Life while Controlling for Psychological Distress*

|  |  | 95% CI | |  |
| --- | --- | --- | --- | --- |
| *Between-Person Effects* | B | LL | UL | p |
| Relative Deprivation ⟷ Meaning in Life | -0.37 | -0.38 | -0.35 | <.001 |
| Relative Deprivation ⟷ Physical Health | -0.38 | -0.39 | -0.37 | <.001 |
| Relative Deprivation ⟷ Psychological Distress | 0.28 | 0.28 | 0.29 | <.001 |
| Meaning in Life ⟷ Physical Health | 0.40 | 0.39 | 0.41 | <.001 |
| Meaning in Life ⟷ Psychological Distress | -0.35 | -0.35 | -0.34 | <.001 |
| Physical Health ⟷ Psychological Distress | -0.29 | -0.29 | -0.28 | <.001 |

|  |  |  |  | 95% CI | |  |
| --- | --- | --- | --- | --- | --- | --- |
| Outcome_T_ | Predictor_T-1_ | *B* | *SE* | *LL* | *UL* | *p* |
| Relative | Meaning in life | -0.03 | 0.01 | -0.04 | -0.012 | .001 |
| Deprivation | Self-rated health | -0.007 | 0.01 | -0.02 | 0.006 | .380 |
|  | Relative deprivation | 0.12 | 0.01 | 0.108 | 0.13 | <.001 |
|  | Psychological distress | 0.03 | 0.01 | 0.00 | 0.05 | .052 |
| Meaning | Meaning in life | 0.11 | 0.01 | 0.1 | 0.12 | <.001 |
|  | Self-rated health | 0.009 | 0.01 | -0.002 | 0.02 | .118 |
|  | Relative deprivation | -0.007 | 0.004 | -0.01 | -0.001 | .051 |
|  | Psychological distress | -0.10 | 0.01 | -0.13 | -0.08 | <.001 |
| Self-rated | Meaning in life | 0.004 | 0.01 | -0.01 | 0.01 | .413 |
| health | Self-rated health | 0.13 | 0.01 | 0.11 | 0.13 | <.001 |
|  | Relative deprivation | -0.002 | 0.003 | -0.01 | 0.004 | .606 |
|  | Psychological distress | -0.03 | 0.01 | -0.04 | -0.01 | .006 |
| Psychological | Meaning in life | -0.01 | 0.003 | -0.02 | -0.01 | <.001 |
| Distress | Self-rated health | -0.01 | 0.003 | -0.02 | -0.01 | <.001 |
|  | Relative deprivation | 0.004 | 0.002 | 0.00 | 0.01 | .056 |
|  | Psychological distress | 0.1 | 0.006 | 0.09 | 0.11 | <.001 |

*Note*. *n* = 58,742. Model fit indices: χ2 (70) = 1691.62, p < .001; CFI = 0.996, RMSEA = 0.020 [0.019, 0.021], p > .999, SRMR = 0.019.

**Supplemental Table 5**

*Results of RI-CLPM for Belonging while Controlling for Psychological Distress*

|  |  | 95% CI | |  |
| --- | --- | --- | --- | --- |
| Between-Person Effects | B | LL | UL | p |
| Relative Deprivation ⟷ Belonging | -0.38 | -0.39 | -0.37 | <.001 |
| Relative Deprivation ⟷ Physical Health | -0.37 | -0.38 | -0.36 | <.001 |
| Relative Deprivation ⟷ Psychological Distress | 0.27 | 0.26 | 0.28 | <.001 |
| Belonging ⟷ Physical Health | 0.36 | 0.35 | 0.37 | <.001 |
| Belonging ⟷ Psychological Distress | -0.32 | -0.33 | -0.32 | <.001 |
| Physical Health ⟷ Psychological Distress | -0.28 | -0.29 | -0.27 | <.001 |

|  |  |  |  | 95% CI | |  |
| --- | --- | --- | --- | --- | --- | --- |
| Outcome_T_ | Predictor_T-1_ | *B* | *SE* | *LL* | *UL* | *p* |
| Relative | Belonging | -0.01 | 0.005 | -0.02 | 0.00 | .21 |
| Deprivation | Self-rated health | -0.01 | 0.005 | -0.02 | 0.00 | .032 |
|  | Relative deprivation | 0.17 | 0.004 | 0.16 | 0.17 | <.001 |
|  | Psychological distress | 0.04 | 0.009 | 0.02 | 0.05 | <.001 |
| Belonging | Belonging | 0.11 | 0.004 | 0.11 | 0.12 | <.001 |
|  | Self-rated health | 0.02 | 0.003 | 0.01 | 0.02 | <.001 |
|  | Relative deprivation | -0.01 | 0.002 | -0.01 | -0.003 | .001 |
|  | Psychological distress | -0.09 | 0.006 | -0.10 | -0.08 | <.001 |
| Self-rated | Belonging | 0.01 | 0.003 | 0.01 | 0.02 | .001 |
| health | Self-rated health | 0.18 | 0.004 | 0.17 | 0.19 | <.001 |
|  | Relative deprivation | -0.01 | 0.002 | -0.01 | -0.001 | .019 |
|  | Psychological distress | -0.04 | 0.006 | -0.05 | -0.03 | <.001 |
| Psychological | Belonging | -0.02 | 0.002 | -0.02 | -0.02 | <.001 |
| Distress | Self-rated health | -0.02 | 0.002 | -0.02 | -0.01 | <.001 |
|  | Relative deprivation | 0.01 | 0.001 | 0.003 | 0.01 | <.001 |
|  | Psychological distress | 0.15 | 0.004 | 0.15 | 0.16 | <.001 |

*Note*. *n* = 66,221. Model fit indices: χ2 (550) = 4206.94, p < .001; CFI = 0.994, RMSEA = 0.010 [0.010, 0.010], p > .999, SRMR = 0.024.

**Supplemental Table 6**

*Results of an RI-CLPM with AR2s for Gratitude*

|  |  |  | 95% CI | |  |
| --- | --- | --- | --- | --- | --- |
| *Between-Person Effects* | *B* | *SE* | *LL* | *UL* | *p* |
| **Relative Deprivation ⟷ Gratitude** | **−0.27** | **0.01** | **[−.28,** | **−0.26]** | **<.001** |
| Relative Deprivation ⟷ Physical Health | **−**0.37 | 0.01 | [**−**0.39, | **−**0.36] | <.001 |
| **Gratitude ⟷ Physical Health** | **0.25** | **0.00** | **[0.24,** | **0.25]** | **<.001** |

| *Within-Person Effects* |  |  |  | 95% CI | |  |
| --- | --- | --- | --- | --- | --- | --- |
| Outcome_T_ | Predictor_T-1_ | *B* | *SE* | *LL* | *UL* | *p* |
| Relative deprivation | Gratitude | **−**0.02 | 0.01 | [**−**0.03, | 0.00] | .094 |
|  | Self-rated health | **−**0.02 | 0.01 | [**−**0.04, | **−**0.01] | .011 |
|  | Relative deprivation | 0.17 | 0.01 | [0.15, | 0.20] | <.001 |
|  | Relative deprivation_T-2_ | 0.05 | 0.01 | [0.03, | 0.07] | <.001 |
| Gratitude | Gratitude | 0.09 | 0.01 | [0.07, | 0.11] | <.001 |
|  | Gratitude _T-2_ | 0.03 | 0.01 | [0.01, | 0.05] | .002 |
|  | Self-rated health | 0.01 | 0.01 | [0.00, | 0.02] | .007 |
|  | **Relative deprivation** | **−0.01** | **0.00** | **[−0.02,** | **−0.01]** | **<.001** |
| Self-rated health | Gratitude | 0.00 | 0.01 | [**−**0.01, | 0.01] | .811 |
|  | Self-rated health | 0.22 | 0.02 | [0.19, | 0.25] | <.001 |
|  | Self-rated health _T-2_ | 0.08 | 0.01 | [0.06, | 0.10] | <.001 |
|  | Relative deprivation | **−**0.01 | 0.00 | [**−**0.01, | 0.00] | .142 |

*Note*. *n* = 58,741. Paths in bold represent statistically significant predicted pathways. Model fit indices: χ^2^_(36)_ = 286.61, *p* < .001; CFI = 0.999, RMSEA = 0.011 [0.010, 0.012], *p* > .999, SRMR = 0.016.

**Supplemental Table 7**

*Results of a multiple-indicator RI-CLPM for Gratitude*

|  |  |  | 95% CI | |  |
| --- | --- | --- | --- | --- | --- |
| *Between-Person Effects* | *B* | *SE* | *LL* | *UL* | *p* |
| **Relative Deprivation ⟷ Gratitude** | **−0.30** | **0.01** | **[−0.31,** | **−0.29]** | **<.001** |
| Relative Deprivation ⟷ Physical Health | **−**0.47 | 0.01 | [**−**0.49, | **−**0.45] | <.001 |
| **Gratitude ⟷ Physical Health** | **0.29** | **0.01** | **[0.28,** | **0.30]** | **<.001** |

| *Within-Person Effects* |  |  |  | 95% CI | |  |
| --- | --- | --- | --- | --- | --- | --- |
| Outcome_T_ | Predictor_T-1_ | *B* | *SE* | *LL* | *UL* | *p* |
| Relative deprivation | Gratitude | **−**0.07 | 0.05 | [**−**0.17, | 0.03] | .188 |
|  | Self-rated health | **−**0.04 | 0.03 | [**−**0.10, | 0.02] | .215 |
|  | Relative deprivation | 0.37 | 0.04 | [0.30, | 0.44] | <.001 |
| Gratitude | Gratitude | 0.20 | 0.04 | [0.13, | 0.27] | <.001 |
|  | Self-rated health | 0.02 | 0.02 | [**−**0.01, | 0.05] | .179 |
|  | **Relative deprivation** | **−0.03** | **0.01** | **[−0.05,** | **−0.01]** | **.004** |
| Self-rated health | Gratitude | **−**0.11 | 0.04 | [**−**0.19, | **−**0.04] | .003 |
|  | Self-rated health | 0.52 | 0.04 | [0.45, | 0.60] | <.001 |
|  | Relative deprivation | **−**0.02 | 0.01 | [**−**0.04, | 0.01] | .254 |

*Note*. *n* = 58,741. Paths in bold represent statistically significant predicted pathways. Model fit indices: χ^2^_(419)_ = 5703.904, *p* < .001; CFI = 0.989, RMSEA = 0.015 [0.014, 0.015], p > .999, SRMR = 0.029.

**Supplemental Table 8**

*Results of an RI-CLPM with AR2s for Meaning in Life*

|  |  |  | 95% CI | |  |
| --- | --- | --- | --- | --- | --- |
| *Between-Person Effects* | *B* | *SE* | *LL* | *UL* | *p* |
| **Relative Deprivation ⟷ Meaning in Life** | **−0.35** | **0.01** | **[−0.37,** | **−0.34]** | **<.001** |
| Relative Deprivation ⟷ Physical Health | **−**0.37 | 0.01 | [**−**0.38, | **−**0.36] | <.001 |
| Meaning in Life ⟷ Physical Health | 0.39 | 0.01 | [0.38, | 0.40] | <.001 |

| *Within-Person Effects* |  |  |  | 95% CI | |  |
| --- | --- | --- | --- | --- | --- | --- |
| Outcome_T_ | Predictor_T-1_ | *B* | *SE* | *LL* | *UL* | *p* |
| Relative deprivation | Meaning in life | **−**0.04 | 0.01 | [**−**0.06, | **−**0.02] | <.001 |
|  | Self-rated health | **−**0.02 | 0.01 | [**−**0.03, | **−**0.00] | .042 |
|  | Relative deprivation | 0.17 | 0.01 | [0.15, | 0.20] | <.001 |
|  | Relative deprivation_T-2_ | 0.05 | 0.01 | [0.03, | 0.07] | <.001 |
| Meaning in life | Meaning in life | 0.24 | 0.01 | [0.21, | 0.26] | <.001 |
|  | Meaning in life _T-2_ | 0.11 | 0.01 | [0.09, | 0.13] | <.001 |
|  | Self-rated health | 0.03 | 0.01 | [0.01, | 0.04] | <.001 |
|  | **Relative deprivation** | **−0.01** | **0.00** | **[−0.02,** | **−0.00]** | **.003** |
| Self-rated health | Meaning in life | 0.01 | 0.01 | [0.00, | 0.02] | .011 |
|  | Self-rated health | 0.22 | 0.02 | [0.19, | 0.24] | <.001 |
|  | Self-rated health _T-2_ | 0.08 | 0.01 | [0.06, | 0.10] | <.001 |
|  | Relative deprivation | **−**0.01 | 0.00 | [**−**0.01, | 0.00] | .214 |

*Note*. *n* = 58,741. Model fit indices: χ^2^_(36)_ = 308.21, *p* < .001; CFI = 0.999, RMSEA = 0.011 [0.010, 0.013], *p* > .999, SRMR = 0.016.

**Supplemental Table 9**

*Results of a multiple-indicator RI-CLPM for Meaning in Life*

|  |  |  | 95% CI | |  |
| --- | --- | --- | --- | --- | --- |
| *Between-Person Effects* | *B* | *SE* | *LL* | *UL* | *p* |
| **Relative Deprivation ⟷ Meaning in Life** | **−0.53** | **0.01** | **[−0.55,** | **−0.51]** | **<.001** |
| Relative Deprivation ⟷ Physical Health | **−**0.47 | 0.01 | [−0.49, | −0.45] | <.001 |
| Meaning in Life ⟷ Physical Health | 0.54 | 0.01 | [0.52, | 0.56] | <.001 |

| *Within-Person Effects* |  |  |  | 95% CI | |  |
| --- | --- | --- | --- | --- | --- | --- |
| Outcome_T_ | Predictor_T-1_ | *B* | *SE* | *LL* | *UL* | *p* |
| Relative deprivation | Meaning in life | **−**0.08 | 0.02 | [−0.12, | −0.04] | <.001 |
|  | Self-rated health | **−**0.02 | 0.03 | [−0.08, | 0.04] | .571 |
|  | Relative deprivation_2_ | 0.39 | 0.03 | [0.32, | 0.46] | <.001 |
| Meaning in life | Meaning in life | 0.34 | 0.03 | [0.29, | 0.39] | <.001 |
|  | Self-rated health | 0.03 | 0.03 | [−0.02, | 0.08] | <.001 |
|  | **Relative deprivation** | **−0.05** | **0.02** | **[−0.08,** | **−0.02]** | **.001** |
| Self-rated health | Meaning in life | −0.04 | 0.02 | [−0.07, | −0.01] | .005 |
|  | Self-rated health | 0.50 | 0.04 | [0.42, | 0.59] | <.001 |
|  | Relative deprivation | **−**0.02 | 0.01 | [−0.04, | 0.01] | .230 |

*Note*. *n* = 58,741. Paths in bold represent statistically significant predicted pathways. Model fit indices: χ^2^_(305)_ = 3296.29, *p* < .001; CFI = 0.994, RMSEA = 0.013 [0.013, 0.013], *p* > .999, SRMR = 0.020.

**Supplemental Table 10**

*Results of a RI-CLPM with AR2s for Belonging*

|  |  |  | 95% CI | |  |
| --- | --- | --- | --- | --- | --- |
| *Between-Person Effects* | *B* | *SE* | *LL* | *UL* | *p* |
| **Relative Deprivation ⟷ Belonging** | **−0.37** | **0.01** | **[−0.39,** | **−0.36]** | **<.001** |
| Relative Deprivation ⟷ Physical Health | **−**0.36 | 0.01 | [−0.37, | **−**0.35] | <.001 |
| **Belonging ⟷ Physical Health** | **0.35** | **0.00** | **[0.34,** | **0.36]** | **<.001** |

| *Within-Person Effects* |  |  |  | 95% CI | |  |
| --- | --- | --- | --- | --- | --- | --- |
| Outcome_T_ | Predictor_T-1_ | *B* | *SE* | *LL* | *UL* | *p* |
| Relative deprivation | Belonging | **−**0.01 | 0.01 | [−0.02, | **−**0.00] | .032 |
|  | Self-rated health | **−**0.01 | 0.01 | [−0.02, | **−**0.00] | .008 |
|  | Relative deprivation | 0.20 | 0.00 | [0.20, | 0.21] | <.001 |
|  | Relative deprivation_T-2_ | 0.10 | 0.00 | [0.09, | 0.10] | <.001 |
| Belonging | Belonging | 0.15 | 0.00 | [0.14, | 0.16] | <.001 |
|  | Belonging_T-2_ | 0.07 | 0.00 | [0.07, | 0.08] | <.001 |
|  | Self-rated health | 0.03 | 0.00 | [0.02, | 0.03] | <.001 |
|  | **Relative deprivation** | **−0.01** | **0.00** | **[−0.01,** | **−0.01]** | **<.001** |
| Self-rated health | **Belonging** | **0.01** | **0.00** | **[0.01,** | **0.02]** | **<.001** |
|  | Self-rated health | 0.22 | 0.00 | [0.21, | 0.23] | <.001 |
|  | Self-rated health_T-2_ | 0.10 | 0.00 | [0.09, | 0.11] | <.001 |
|  | Relative deprivation | **−**0.01 | 0.00 | [−0.01, | **−**0.00] | .008 |

*Note*. *n* = 66,221. Model fit indices: χ^2^_(306)_ = 1800.416, *p* < .001; CFI = 0.996, RMSEA = 0.009 [0.008, 0.009], *p* > .999, SRMR = 0.023.

**Supplemental Table 11**

*Results of a multiple-indicator RI-CLPM for Belonging*

|  |  |  | 95% CI | |  |
| --- | --- | --- | --- | --- | --- |
| *Between-Person Effects* | *B* | *SE* | *LL* | *UL* | *p* |
| **Relative Deprivation ⟷ Belonging** | **−0.41** | **0.01** | **[−0.43,** | **−0.40]** | **<.001** |
| Relative Deprivation ⟷ Physical Health | **−**0.46 | 0.01 | [−0.47, | **−**0.44] | <.001 |
| **Belonging ⟷ Physical Health** | **0.41** | **0.01** | **[0.40,** | **0.42]** | **<.001** |

| *Within-Person Effects* |  |  |  | 95% CI | |  |
| --- | --- | --- | --- | --- | --- | --- |
| Outcome_T_ | Predictor_T-1_ | *B* | *SE* | *LL* | *UL* | *p* |
| Relative deprivation | Belonging | **−**0.00 | 0.02 | [−0.04, | 0.03] | .918 |
|  | Self-rated health | 0.01 | 0.02 | [−0.02, | 0.04] | .387 |
|  | Relative deprivation | 0.52 | 0.02 | [0.48, | 0.56] | <.001 |
| Belonging | Belonging | 0.30 | 0.02 | [0.27, | 0.33] | <.001 |
|  | Self-rated health | 0.05 | 0.01 | [0.03, | 0.07] | <.001 |
|  | **Relative deprivation** | **−0.03** | **0.01** | **[−0.04,** | **−0.02]** | **<.001** |
| Self-rated health | **Belonging** | **−0.05** | **0.01** | **[−0.08,** | **−0.03]** | **<.001** |
|  | Self-rated health | 0.57 | 0.02 | [0.54, | 0.60] | <.001 |
|  | Relative deprivation | 0.00 | 0.01 | [−0.01, | 0.01] | .970 |

*Note*. *n* = 66,221. Model fit indices: χ^2^_(2234)_ = 16622.430, *p* < .001; CFI = 0.984, RMSEA = 0.010 [0.010, 0.010], *p* > .999, SRMR = 0.030.

**Table 12**

*Results of a STARTS model for Gratitude*

|  |  |  | 95% CI | |  |
| --- | --- | --- | --- | --- | --- |
| *Correlations Between Stable Traits* | *B* | *SE* | *LL* | *UL* | *p* |
| **Relative Deprivation ⟷ Gratitude** | **−0.22** | **0.02** | **[−0.26,** | **−0.18]** | **<.001** |
| Relative Deprivation ⟷ Physical Health | **−**0.33 | 0.01 | [**−**0.35, | **−**0.30] | <.001 |
| **Gratitude ⟷ Physical Health** | **0.24** | **0.02** | **[0.19,** | **0.28]** | **<.001** |

| *Lagged Autoregressive Trait Effects* | |  |  | 95% CI | |  |
| --- | --- | --- | --- | --- | --- | --- |
| Outcome_T_ | Predictor_T-1_ | *B* | *SE* | *LL* | *UL* | *p* |
| Relative deprivation | Gratitude | **−**0.25 | 0.52 | [**−1**.27, | 0.76] | .627 |
|  | Self-rated health | **−**0.12 | 0.24 | [**−**0.59, | 0.34] | .597 |
|  | Relative deprivation | 0.55 | 0.43 | [**−**0.29, | 1.39] | .200 |
| Gratitude | Gratitude | 0.76 | 0.28 | [0.21, | 1.31] | .007 |
|  | Self-rated health | 0.14 | 0.17 | [**−**0.02, | 0.05] | .408 |
|  | Relative deprivation | −0.05 | 0.09 | [−0.22, | 0.12] | .578 |
| Self-rated health | Gratitude | **−**0.05 | 0.07 | [**−**0.19, | 0.10] | .532 |
|  | Self-rated health | 0.74 | 0.18 | [0.39, | 1.09] | <.001 |
|  | Relative deprivation | **−**0.06 | 0.09 | [**−**0.24, | 0.13] | .563 |

*Note*. *n* = 58,741. Paths in bold represent statistically significant predicted pathways. Model fit indices: χ^2^_(51)_ = 713.474, *p* < .001; CFI = 0.996, RMSEA = 0.015 [0.014, 0.016], p > .999, SRMR = 0.028. Model is based on constrained maximum likelihood estimates with robust standard errors.

**Table 13**

*Results of a STARTS model for Meaning in Life*

|  |  |  | 95% CI | |  |
| --- | --- | --- | --- | --- | --- |
| *Correlations Between Stable Traits* | *B* | *SE* | *LL* | *UL* | *p* |
| **Relative Deprivation ⟷ Meaning in Life** | **−0.29** | **0.01** | **[−0.31,** | **−0.27]** | **<.001** |
| Relative Deprivation ⟷ Physical Health | **−**0.34 | 0.01 | [**−**0.36, | **−**0.31] | <.001 |
| Meaning in Life ⟷ Physical Health | 0.36 | 0.01 | [0.34, | 0.39] | <.001 |

| *Lagged Autoregressive Trait Findings* | |  |  | 95% CI | |  |
| --- | --- | --- | --- | --- | --- | --- |
| Outcome_T_ | Predictor_T-1_ | *B* | *SE* | *LL* | *UL* | *p* |
| Relative deprivation | Meaning in life | **−**0.31 | 0.08 | [**−**0.47, | **−**0.15] | <.001 |
|  | Self-rated health | **−**0.08 | 0.06 | [**−**0.20, | 0.05] | .223 |
|  | Relative deprivation | 0.43 | 0.07 | [0.30, | 0.57] | <.001 |
| Meaning in life | Meaning in life | 0.68 | 0.06 | [0.55, | 0.80] | <.001 |
|  | Self-rated health | 0.05 | 0.03 | [**−**0.02, | 0.11] | .156 |
|  | **Relative deprivation** | **−0.12** | **0.03** | **[−0.17,** | **−0.06]** | **<.001** |
| Self-rated health | Meaning in life | **−**0.00 | 0.03 | [**−**0.07, | 0.06] | .954 |
|  | Self-rated health | 0.74 | 0.06 | [0.61, | 0.86] | <.001 |
|  | Relative deprivation | **−**0.04 | 0.03 | [−0.10, | 0.02] | .160 |

*Note*. *n* = 58,741. Paths in bold represent statistically significant predicted pathways. Model fit indices: χ^2^_(51)_ = 512.874, *p* < .001; CFI = 0.997, RMSEA = 0.012 [0.011, 0.013], *p* > .999, SRMR = 0.019. Model is based on constrained maximum likelihood estimates with robust standard errors.

**Table 14**

*Results of a STARTS model for Belonging*

|  |  |  | 95% CI | |  |
| --- | --- | --- | --- | --- | --- |
| *Correlations Between Stable Traits* | *B* | *SE* | *LL* | *UL* | *p* |
| **Relative Deprivation ⟷ Belonging** | **−0.24** | **0.01** | **[−0.26,** | **−0.22]** | **<.001** |
| Relative Deprivation ⟷ Physical Health | **−**0.32 | 0.01 | [−0.35, | **−**0.30] | <.001 |
| **Belonging ⟷ Physical Health** | **0.28** | **0.01** | **[0.25,** | **0.30]** | **<.001** |

| *Lagged Autoregressive Trait Findings* | |  |  | 95% CI | |  |
| --- | --- | --- | --- | --- | --- | --- |
| Outcome_T_ | Predictor_T-1_ | *B* | *SE* | *LL* | *UL* | *p* |
| Relative deprivation | Belonging | **−**0.15 | 0.02 | [−0.20, | −0.10] | <.001 |
|  | Self-rated health | 0.03 | 0.01 | [0.01, | 0.06] | .012 |
|  | Relative deprivation | 0.81 | 0.02 | [0.77, | 0.85] | <.001 |
| Belonging | Belonging | 0.84 | 0.02 | [0.80, | 0.88] | <.001 |
|  | Self-rated health | 0.04 | 0.01 | [0.01, | 0.06] | .003 |
|  | **Relative deprivation** | **−0.05** | **0.01** | **[−0.07,** | **−0.04]** | **<.001** |
| Self-rated health | **Belonging** | **0.04** | **0.02** | **[0.01,** | **0.08]** | **.017** |
|  | Self-rated health | 0.83 | 0.02 | [0.80, | 0.87] | <.001 |
|  | Relative deprivation | 0.01 | 0.01 | [−0.01, | 0.02] | .347 |

*Note*. *n* = 66,221. Paths in bold represent statistically significant predicted pathways. Model fit indices: χ^2^_(351)_ = 1824.132, *p* < .001; CFI = 0.996, RMSEA = 0.008 [0.008, 0.008], *p* > .999, SRMR = 0.023. Model is based on constrained maximum likelihood estimates with robust standard errors
